# Early impairment of contractility reserve in patients with insulin resistance in comparison with healthy subjects

**DOI:** 10.1186/1475-2840-12-66

**Published:** 2013-04-16

**Authors:** Christian Cadeddu, Silvio Nocco, Davide Piano, Martino Deidda, Efisio Cossu, Marco Giorgio Baroni, Giuseppe Mercuro

**Affiliations:** 1 Department of Medical Sciences “M. Aresu”, University of Cagliari, SS 554, Km 4.500, Monserrato, Cagliari, 09042, Italy

**Keywords:** Insulin resistance, Stress echocardiography, Contractility reserve

## Abstract

**Background:**

Insulin resistance (IR) is currently considered a crucial cardiovascular (CV) risk factor, which seems to play a dominant role in the evolution toward cardiac and vascular impairment. Early IR-induced cardiac dysfunction can be assessed by Doppler-derived myocardial systolic strain rate (SR) index, measured at baseline and after dobutamine stress echocardiography (DSE).

**Methods:**

Thirty IR patients (HOMA-IR = 7 ± 5.2, age 52.6 ± 2.1 years), and 20 healthy, age and sex matched controls were studied. IR had been diagnosed in all patients in the 3 months preceding the study. Dobutamine echocardiography was performed in all subjects to exclude ischemic heart disease, and left ventricular contractile reserve (LVCR) was then assessed. LVCR was evaluated as an increase in the peak of an average longitudinal SR, measured in the basal and mid segments of 2 and 4 chamber ventricular walls.

**Results:**

No significant differences between the 2 groups were revealed by baseline echocardiography. In contrast, after DSE a significant decrease of Delta SR was found in the IR group in comparison to the controls (0.54 ± 0.31 s^−1^*vs* 1.14 ± 0.45 s^−1^; p < 0.0001).

**Conclusions:**

Our results show that IR, even if isolated and arising within a short time period, not only represents the initial phase of future diabetes, but may adversely affect heart function, as evidenced by the depressed LVCR. Our data strengthen the need for attention to be paid to IR state and for an early therapeutic approach.

## Background

Diabetes mellitus has been shown to induce cardiac contractile dysfunction, which results from metabolic derangements and may evolve into a specific cardiomyopathy [1]. It has also been well established that the onset of type 2 diabetes mellitus is preceded by a variable period of abnormal glucose homeostasis, characterized by progressive insulin resistance (IR) and impairment of β-cell function [2].

Individuals with IR are at high risk of adverse cardiovascular events (myocardial infarction, stroke, cardiovascular death) later in life. A high prevalence of heart failure is associated with IR [3,4], and epidemiological evidence suggests that the metabolic disorder precedes cardiac decompensation.

Recently, IR has been shown to predict the subsequent development of heart failure, regardless of all known risk factors, including diabetes mellitus [5,6].

On the basis of this evidence, the existence of an “insulin-resistant cardiomyopathy” has been proposed [7] and increased interest has been directed to the specific cellular mechanisms by which this metabolic disorder can lead to cardiac structural and functional abnormalities [8].

With regard to the causal relationship between IR and cardiac function, the availability of epidemiological and clinical findings are not matched by an equal knowledge of the underlying mechanisms: alterations in hemodynamics, plasma volume, neurohormonal status, and myocardial substrate metabolism, all appear to contribute to these changes [7]. In relation to the high probability that the pre-diabetic condition can cause myocardial impairment, it would be extremely valuable to detect early contractile function changes in subjects affected by IR alone, prior the development of the metabolic syndrome as well as from overt cardiovascular disease.

Dobutamine stress echocardiography (DSE), widely used in patients with coronary artery disease or dilated cardiomyopathy, enables evaluation of left ventricular contractile reserve (LVCR) [9]. In particular, the assessment of longitudinal LVCR has proved useful to identify early myocardial dysfunction in patients with type 2 diabetes [10,11].

Based on the hypothesis that IR might be an independent factor responsible for the development of impaired systolic function, the LVRC of a population of individuals <55 years, whose IR had been diagnosed in the 3 months before the study, was compared to that of a control group matched for age, gender and body mass index (BMI).

## Methods

Thirty-two patients (15 male/17 female), aged <55 years (52.6 ± 2.1 years), were consecutively selected from a population of individuals screened at the Diabetic Centre of our University Hospital and enrolled on the study. All patients presented impaired glucose tolerance (IGT) which had been identified within the previous 3 months, and/or impaired fasting glucose (IFG), and all were affected by IR, calculated in accordance with the Homeostasis Model Assessment (HOMA) index and defined according to the values of Bonora et al. [12]. The patients were referred to our stress echo laboratory as they reported a family history of ischemic heart disease. Twenty age, gender and body mass index matched normoglycemic subjects (HOMA <2.8; 9 male/11 female), with no structural heart defects or evidence of ischemic heart disease were also enrolled as controls.

Inclusion criteria for both IR patients and controls were: age 20–55 years, echocardiographic left ventricular ejection fraction (LVEF) ≥55% and absence of echocardiographic wall motion abnormalities, normal hepatic and renal function (bilirubin ≤1.5 mg/dl, creatinine ≤2.0 mg/dl). Exclusion criteria were diabetes, smoking, hypertension with LV hypertrophy, obesity (overweight was tolerated with a BMI <29.9), moderate to severe heart valve disease, atrial fibrillation or severe arrhythmias. The presence of dyslipidaemia or hypertension without left ventricular (LV) hypertrophy was admitted, provided that only one of these risk factors was present in any single individual in combination with IR. No patient was on medication that could influence glucose metabolism or cardiac function. The present study was approved by the Ethical Committee of our University Hospital and informed written consent was obtained from all participants.

At enrolment, physical examination, 12-lead electrocardiogram, M-mode, 2D and Doppler-echocardiography, and complete blood chemistry were performed in all subjects.

### Conventional echocardiography and Tissue Doppler Imaging (TDI)

All participants were instructed on the medical environment and instrumentation before testing. Echocardiographic images were recorded using a TDI imaging and raw data acquisition system (Toshiba Aplio; Toshiba Corp., Tochigi, Japan). LVEF was obtained from the apical 4- and 2-chamber views according to Simpson’s rule and was considered abnormal when <55%. Pulsed Wave Doppler (PWD) examination was carried out of the LV inflow from the 4-chamber view with the sample volume placed between the mitral leaflet tips and the early (E) and late (A) diastolic peak velocities; E deceleration time (DecT) was measured and E/A ratio was derived.

Longitudinal function was assessed using pulsed TDI at mitral annulus, placing the sample volume in the basal segment of the interventricular septum (IVS) from the apical 4-chamber view; peak velocities in systole (Sm), early (Em) and late (Am) diastole were measured. LV longitudinal function at baseline and after DSE was evaluated offline from raw data (TDI-Q, Toshiba); longitudinal strain (Σ) and strain rate (SR) (average from basal and mid segments of 4 chamber and 2 chamber view) were also quantified based on TDI imaging. All the examinations on each patient were carried out by a single experienced echocardiographer. A simultaneous electrocardiographic trace was also obtained. To reduce inter-observer variability, all echocardiographic data were randomly read by a second experienced observer and an average value for each measurement was calculated. Reproducibility of TDI parameters in our laboratory had been previously documented [13].

### DSE

Dobutamine was administered according to standard 3-min incremental protocol (5 to 40 μg/kg/min), and atropine was injected in patients who had not achieved 85% of their maximal heart rate (1 mg, with increments of 0.25 mg until the target heart rate) [14]. TDI of the 4 and 2 chamber view was recorded at rest and at peak dose. The 12-lead ECG (Mortara instruments, inc.) was monitored throughout, and blood pressure (BP) recorded at rest and at 3-min intervals during infusion and recovery. End points of the stress protocol were completion of the protocols, progressive or severe chest pain, severe ventricular arrhythmias, systolic BP of 240 mm Hg, diastolic BP of 100 mm Hg, or severe adverse effects.

Tissue Doppler-derived velocities, SR, and Σ were measured off line from raw data. According to the 16-segment model of the 4 tomographic LV views, regional wall motion was scored as normal (1), hypokinetic (2), akinetic (3), and dyskinetic (4), and wall motion score index (WMSI) was derived by two experienced observers blinded to the patient’s clinical data.

### Statistical analysis

With regards the anthropometric and clinical characteristics of the two groups, continuous variables were compared with ANOVA, and categorical variables were compared with the Fisher’s exact test. Differences in echocardiographic parameters were also evaluated using ANOVA. A two-tailed value of p < 0.05 was considered statistically significant. Data are presented as mean ± SD.

Receiver operating characteristic (ROC) analysis was used to assess the best cut-off value of Delta SR to predict myocardial contractile reserve.

A measure of the area under the ROC curve was calculated. Sensitivity, specificity, and accuracy calculations were performed according to standard definitions. The 95% CIs were calculated and the individual intervals were compared.

## Results

Two of the 32 IR patients were excluded from the study as they were positive for ischemia at DSE. They then underwent coronary angiography which confirmed significant coronary stenosis. The clinical and laboratory data of the remaining 30 patients and controls are summarized in Table 1. The subjects of both groups were on average overweight, with IR patients showing a significantly higher BMI than controls (28.8 ± 5.5 kg/m2 vs 26 ± 3.5 kg/m2; p < 0.05). None of the investigated individuals were obese. A HOMA >2.77 as anticipated, was a criterion for inclusion in the study for all patients [12]. Conversely, a HOMA ≤2.77 and fasting glucose within the normal range was demonstrated in all control group subjects. No differences were observed between the two groups of individuals in terms of arterial hypertension with no LV hypertrophy and hyperlipidaemia. In agreement with the exclusion criteria, no patient or control subject was affected by both of the 2 risk factors (Table 1).

**Table 1:** Clinical and laboratory data

**Parameter**	**IR patients**	**Controls**	***p***
**Age (yrs)**	52.6 ± 2.1	51.8 ± 2.0	ns
**Weight (kg)**	81.3 ± 14	72 ± 14	ns
**BMI (kg/m**^**2**^**)**	27.3 ± 1.4	26 ± 3.0	<0.05
**Waist circumference (cm)**	98 ± 6.2	92 ± 5.3	ns
**Fasting glucose (mg/dl)**	114 ± 13	92 ± 11	<0.01
**Homa IR**	7 ± 5.2	1.4 ± 0.8	<0.01
**Total cholesterol (mg/dl)**	204 ± 16	184 ± 26	ns
**HDL (mg/dl)**	52 ± 11	57 ± 9	ns
**LDL (mg/dl)**	128 ± 14	117 ± 18	ns
**Triglycerides (mg/dl)**	149 ± 50	111 ± 38	ns
**Smoking**	None	None	--
**Hypertension (%) no LVH**	27%	35%	ns

*Abbreviations: BMI* Body mass index, *LVH* left ventricle hypertrophy.

All patients and controls showed a normal baseline ECG, as well as normal LV dimensions, systolic function, and ventricular mass (Table 2). The baseline longitudinal systolic function provided by TD imaging (Sm wave) was also within normal range in both groups. Finally, longitudinal Σ and SR at rest were comparable in patients and controls (Table 3).

**Table 2:** Transthoracic conventional echocardiographic and TDI parameters

**Parameter**	**Insulin resistance**	**Controls**	***p***
**EDD (mm)**	47.86 ± 4.56	47.01 ± 5.87	ns
**EDV (ml)**	80.62 ± 20.20	70.65 ± 4.26	ns
**LAA (cm**^**2**^**)**	18.63 ± 3.09	18.63 ± 4.32	ns
**LVM (g/m**^**2**^**)**	63.83 ± 13.27	57.56 ± 11.21	ns
**LVEF (%)**	66 ± 5.1	65 ± 4.9	ns
**E/A ratio**	0.92 ± 0.22	1.02 ± 0.27	ns
**DecT (sec)**	0.22 ± 0.06	0.20 ± 0.04	ns
**Sm (cm/sec)**	46.9 ± 7.7	52.9 ± 6.2	ns
**Em (m/sec)**	7.9 ± 1.9	8.2 ± 1.6	ns
**E/Em**	9.9 ± 2.56	9.8 ± 2.01	ns

*Abbreviations: EDD* end diastolic diameter, *EDV* end diastolic volume, *LAA* left atrium area, *LVM* left ventricular mass, *LVEF* Left Ventricular Ejection Fraction, *E/A* early and late diastolic peak velocity ratio, *DecT* E wave deceleration time, *S*_*m*_ TD systolic peak velocity, *E*_*m*_ TD early diastolic peak velocity, *E/E* early PWD and *TD* diastolic peak velocity ratio.

**Table 3:** Longitudinal Σ (%) and SR (s-1)

	**Controls**	**Insulin resistance**
	*Strain rate imaging*	
*Basal*		
Σ (%)	23.9 ± 6.13	24.5 ± 8.22
SR (sec^−1^)	1.74 ± 0.32	1.91 ± 0.29
*High dose dobutamine*		
Σ (%)	26.7 ± 4.32	23.6 ± 5.1
SR (sec^−1^)	2.87 ± 0.26*	2.45 ± 0.21*
*Δ Stress*		
Σ (%)	+2.2 ± 1.8 (+9%)	−0.9 ± 1.7 (−4%)
SR (sec^−1^)	1.14 ± 0.45 (+66%)*	0.54 ± 0.31 (+27%)*

*Abbreviations: SR* Strain Rate, *Σ* Strain. *P < 0.0001.

**Figure 1:**
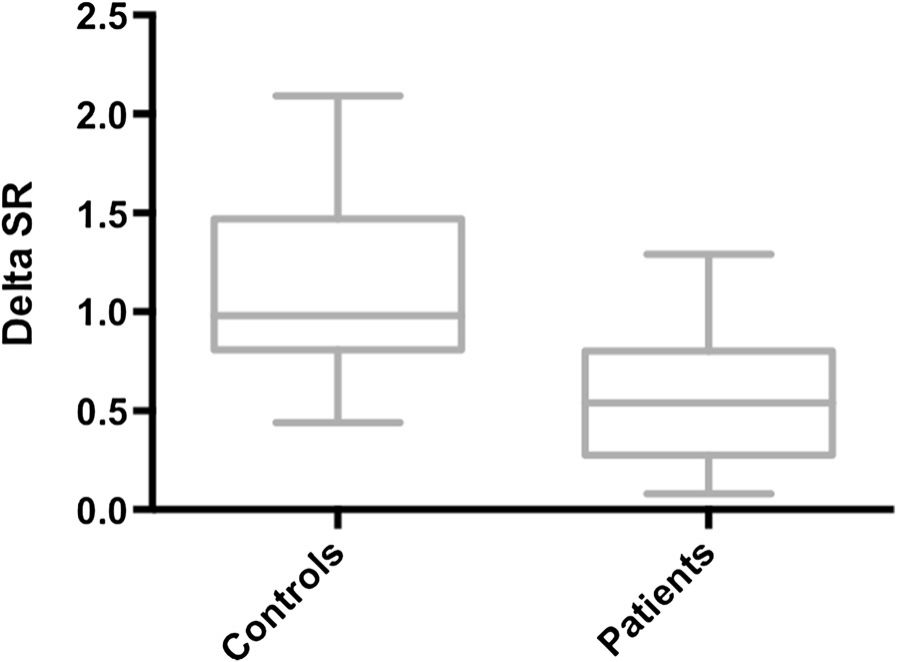
SR Delta SR values obtained in patients and in healthy controls.

All participants underwent DSE (see Methods), which was generally well tolerated. Minor predictable inconveniences, such as palpitations and systolic hypotension occurred, but in no case did discontinuation of the test result. With regard to the outcome of the pharmacological stress test, IR patients showed reduced longitudinal SR at the peak of drug administration as compared to controls (2.45 ± 0.21 sec^−1^*vs* 2.87 ± 0.26 sec^−1^, p < 0.0001, Table 3). Moreover, Delta SR between peak and basal values was significantly reduced in IR patients when compared with controls (Figure 1; 0.54 ± 0.31 sec^−1^*vs* 1.14 ± 0.45 sec^−1^, p < 0.0001, Table 3), thereby revealing reduced LVCR.

**Figure 2:**
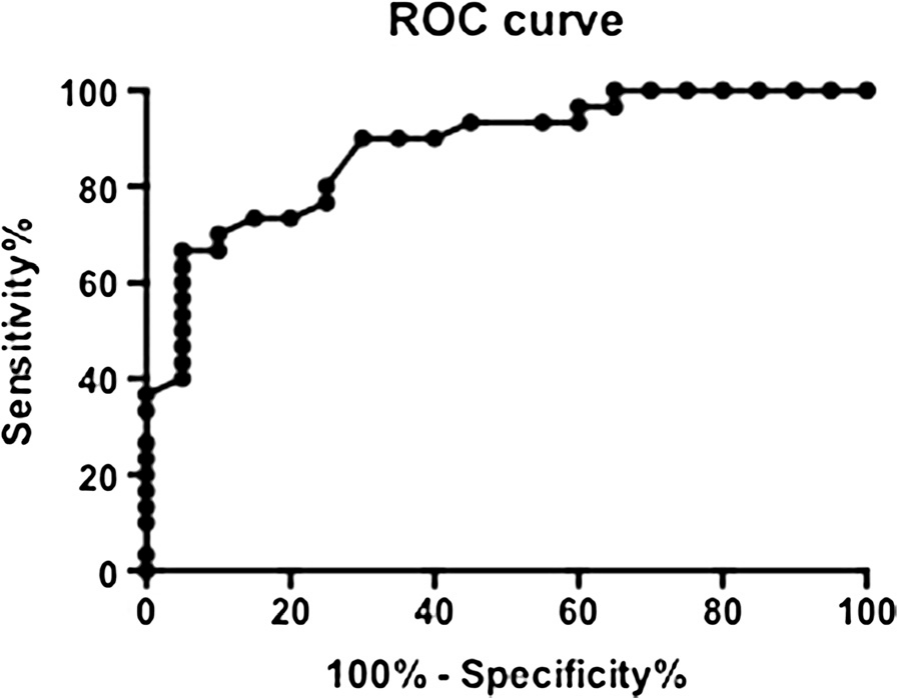
**ROC analysis of stress–rest difference in the mean value of peak systolic SR.** [Area under the curve 0.879 (95% CI 0.79–0.97)].

Using ROC analysis, a value of 0.85 sec^−1^ obtained as a stress–rest difference in the mean value of peak systolic SR was the best cut-off value to diagnose myocardial contractile reserve [area under the curve 0.879 (95% CI 0.79–0.97), sensitivity 90% (95% CI 73–98), specificity 70% (95% CI 46–88)] (Figure 2).

There were no changes in the Σ (Table 3), due to the influence exercised on this parameter by stroke volume, which tends to be reduced after dobutamine, for the increase in heart rate and the resulting reduction in diastolic filling.

## Discussion

The present study examined baseline cardiac performance and LVCR in middle-aged individuals (mean age 52.6 ± 2.1 years) with recent recognition (2.3 ± 0.8 months) of IR, without diabetes and free from cardiovascular clinical disease. They were compared with age and gender matched subjects with normal glucose tolerance. We found that: *i.* baseline echocardiography LV mass, dimensions and systolic function (EF%) were comparable in insulin-resistant individuals and controls; *ii.* longitudinal Σ and SR at rest were similar between the 2 groups; *iii.* subjects with IR showed a reduced increase in longitudinal SR values (Delta SR) at DSE in comparison with controls, revealing reduced LVCR. These results suggest that IR, even shortly after onset, is associated with cardiac dysfunction, detectable under conditions of greater functional effort.

### IR and myocardial characteristics

The period preceding overt of type 2 diabetes mellitus is characterized by a state of IR and worsened β-cell function, which is clearly evident in subjects with IGT and/or IFG. In turn, IR has been independently associated with magnetic resonance measures of LV mass and LV mass to end-diastolic volume ratio, suggesting involvement in concentric LV remodelling [15]. Abnormal glucose homeostasis was found correlated with impaired LV diastolic function [16], and this relationship has been shown to be independent of blood pressure, LV geometry, total plasma lipids and obesity [17]. In a large community-based sample, LV mass and wall thickness increased across categories of worsening glucose tolerance [18]. Moreover, cardiometabolic profile and inflammation markers have been shown to be more severely altered in men and women with both IFG and IGT compared with those with IFG alone. These individuals, in the absence of hypertension, were seen to have a 10-fold greater probability of preclinical cardiovascular disease [19]. IR was also seen to be an independent predictor of atherosclerosis plaque progression in patients with coronary heart disease in both the diabetic and non-diabetic population [20].

Despite these important premises, our IR patients showed conventional echocardiographic parameters within normal limits at baseline. In particular, no increase in myocardial mass was observed, in comparison with controls. The absence of LV remodelling in these subjects can be ascribed to the very recent emergence of IR, and that the blood pressure was well controlled in most cases. On the contrary, it is well documented that long-standing IR, not countered by any therapeutic measure, is co-responsible for the development of LV hypertrophy, as well as for myocardial fibrosis, a crucial modification in the pathogenesis of a cardiomyopathy [21].

### IR and myocardial energetic metabolism

It is becoming increasingly evident that minor abnormalities occur in the insulin-resistant state that precedes the manifestation of overt type 2 diabetes, although IR patients were mostly investigated in the presence of co-morbidities and/or a long time after the initial diagnosis [7,8]. IR can lead to myocardial injury by contributing to lipotoxicity, sympathetic up-regulation, inflammation, oxidative stress, and fibrosis. The existence of an insulin-resistant cardiomyopathy, which is characterized by inefficient energy metabolism [7], has been demonstrated in animal models. In sucrose-fed rats, serial echocardiographic assessments revealed that early abnormalities in diastolic function, followed by late systolic dysfunction, were associated with IR. A concurrent depressed sarcoplasmic reticulum function was demonstrated by a significant reduction in Ca (2+) uptake [1].

In the IR patients in this study, both indices of diastolic function and highly sensitive parameters of intrinsic myocardial function, such as longitudinal Σ and SR, were comparable at rest to those of healthy controls. As previously stated, this baseline myocardial efficiency can be attributed to the very recent diagnosis of IR. Moreover, the relatively young age (less than 55 years) of our patients, contributed to minimize the effects of aging on cardiac performance.

### IR and inotropic reserve

Assuming that patients with recent IR onset are carriers of an unapparent myocardial dysfunction at baseline rest, their levels of functional coronary reserve were investigated by DSE. Indeed, the provocative test revealed an initial but significant contractile dysfunction in these subjects, denoted by a smaller inotropic response to dobutamine in comparison to controls. The presence of minor changes in systolic performance documents a functional IR-related cardiomyopathy, distinct from the diabetic morphological type, characterized by macroscopic changes, such as hypertrophy.

The recognition of an early decline of LVCR in middle-aged individuals with isolated IR of recent onset is the original aspect of this study. In previous studies on this topic, older populations were recruited, IR was accompanied by comorbidities, especially obesity, and, above all, glucose intolerance was manifested over much longer time periods [5,7,8,22,23].

In a recent study, longitudinal contractile reserve was found significantly lower in pre-diabetic subjects during exercise. They were aged 36–76 years, suffered from Metabolic Syndrome and were hypertensive [24].

Although our results suggest that IR may act via a direct and independent mechanism, we can only hypothesize the pathophysiological basis of this effect. At the myocardial level, insulin inhibits NO production [25]; consequently, an insufficient response to the hormone may determine reduced NO release, with potential endothelial dysfunction. More recently, IR that has been reported in association with exercise intolerance in heart failure patients has been partly attributed to reduced coronary flow reserve [26]. Moreover, alteration of the myocardial energetic metabolism caused by IR can lead to ATP production impairment [27-30]. This dysfunction may become significant under conditions of increased energy demand, such as during catecholaminergic stress. These mechanisms, masked in basal conditions and disclosed by pharmacological stress, should have resulted in contractile reserve reduction in our IR individuals.

## Conclusions

It is well recognized that IR can contribute to an increased risk of coronary heart disease [30], it is highly prevalent in non-ischemic heart failure, and it is a common co-morbidity in congestive heart failure [6], where IR predicts the development and independently defines a worse prognosis [7]. In accordance with this clinical evidence, our results recommend the greatest attention be paid to IR state from its initial appearance and support an early therapeutic approach to this threatening metabolic defect. We recently showed that early metformin treatment in IR patients can improve endothelial function and even cardiopulmonary performance in those subjects with higher HOMA-IR [31,32].

Moreover, a non-invasive, repeatable assessment of coronary circulatory dysfunction, like the one employed in this study, may enable us to monitor myocardial response to corrective actions with drugs, diet and physical activity.

Further extensive and prolonged studies are required to confirm the validity of the approach used in this study as a tool for image-guided and personalized diabetic preventive care. Our results show that IR, even if isolated and arising within a short time period, not only represents the initial phase of future diabetes, but may adversely affect heart function, as evidenced by the depressed LVCR. Our data strengthen the need for attention to be paid to IR state and for an early therapeutic approach.

## Abbreviations

IR: Insulin resistance; CV: Cardiovascular; SR: Strain rate; DSE: Dobutamine stress echocardiography; LVCR: Left ventricular contractile reserve; IGT: Impaired glucose tolerance; IFG: Impaired fast glucose; HOMA: HOmeostasis model assessment; BMI: Body mass index; LV: Left ventricule; LVEF: Left ventricule ejection fraction; PWD: Pulsed wave doppler; TDI: Tissue doppler imaging; IVS: Interventricular septum; Σ: Strain; BP: Blood pressure; WMS: Wall motion score; WMSI: Wall motion score index.

## Competing interests

The authors declare that they have no competing interests.

## Authors’ contributions

CC, Study design, manuscript writing. SN, Study design, Data collection. DP, Echocardiography and clinical examination. MD, Echocardiography, Data analysis. EC, Patients recruitment, study design. MGB, Study design, manuscript writing. GM, Study Design, Manuscript writing, Data interpretation. All authors read and approved the final manuscript.
